# Design of a Novel Axial Gas Pulses Micromixer and Simulations of its Mixing Abilities via Computational Fluid Dynamics

**DOI:** 10.3390/mi10030205

**Published:** 2019-03-23

**Authors:** Florian Noël, Christophe A. Serra, Stéphane Le Calvé

**Affiliations:** 1 ICPEES UMR 7515, Université de Strasbourg/CNRS, F-67000 Strasbourg, France; florian.noel@etu.unistra.fr; 2 In’Air Solutions, 25 rue Becquerel, 67087 Strasbourg, France; 3 Institut Charles Sadron (ICS) UPR 22, Université de Strasbourg/CNRS, F-67000 Strasbourg, France; ca.serra@unistra.fr

**Keywords:** gas mixing, pulsed flow, modular micromixer, multi-stage micromixer, modelling

## Abstract

Following the fast development of microfluidics over the last decade, the need for methods for mixing two gases in flow at an overall flow rate ranging from 1 to 100 NmL·min^−1^ with programmable mixing ratios has been quickly increasing in many fields of application, especially in the calibration of analytical devices such as air pollution sensors. This work investigates numerically the mixing of pure gas pulses at flow rates in the range 1–100 NmL·min^−1^ in a newly designed multi-stage and modular micromixer composed of 4 buffer tanks of 300 µL each per stage. Results indicate that, for a 1 s pulse of pure gas (formaldehyde) followed by a 9 s pulse of pure carrier gas (air), that is a pulses ratio of 1/10, an effective mixing up to 94–96% can be readily obtained at the exit of the micromixer. This is achieved in less than 20 s for any flow rate ranging from 1 to 100 NmL·min^−1^ simply by adjusting the number of stages, 1 to 16 respectively. By using an already diluted gas bottle containing 100 ppm of a given compound in an inert gas same as the carrier gas, concentrations ranging from 10 to 90 ppm should be obtained by adjusting the pulses ratio between 1/10 and 9/10 respectively.

## 1. Introduction

The homogenization of a gaseous chemical mixture is of particular interest in many, sometimes complex processes with multiple and varied applications [1,2,3,4,5,6]. This is particularly the case for the generation of gas mixtures of known concentrations for supplying chemical reactors [7,8] or analytical devices for their calibration [9,10]. Most of the time, the applied flow rates vary from a few litres per minute for gas calibration generators [11,12,13] to several m^3^ per hour for industrial processes [14].

**Figure 1:**
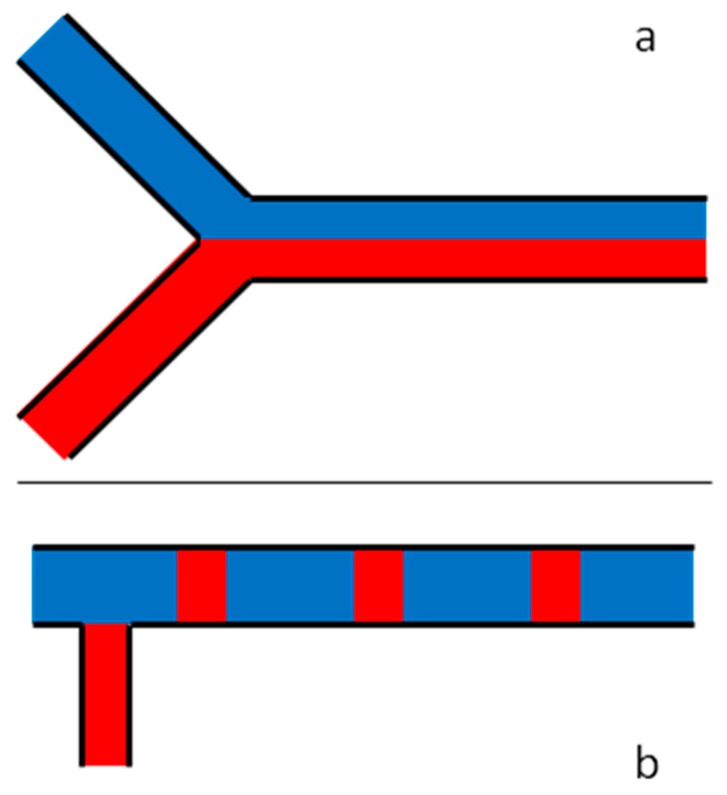
Schematic representation of a two-fluid flow with radial (**a**) or axial (**b**) heterogeneity before diffusion mixing operates.

Today, analytical devices become more and more miniaturized and industrial processes are increasingly using microfluidic devices to improve energy [15] and chemical reactions [16] yields. Thus, low gas flow rates, typically less than 1 NmL·min^−1^, start to be considered. However, some applications still require flow rates over 1 NmL·min^−1^ but lower than few hundreds of NmL·min^−1^. Then, manipulating gas in microchannels has become a way of achieving the miniaturization of many devices in several fields of application. Fluids manipulation implies, *inter alia*, droplets generation [17,18,19], multi-phase flows [20,21] and fluids mixing which is a crucial aspect in several fields of application. Various ways of mixing gas flows presenting a radial heterogeneity (Figure 1a) already exist, going from passive to electronics- or sound-driven mixing techniques [22] but none have been already experimentally used to solve for axial heterogeneity (Figure 1b).

At total flow rates around or lower than a few NmL·min^−1^ (e.g., for air pollution sensors), low mixing ratios become an issue for the usual mixers since they require to control a very small flow rate of the fluid to be diluted (typically below 1 NmL·min^−1^). This implies to rely on highly accurate flow controllers which is a real issue for gas flows technologies. However, this issue is negated by the pulsed flow mixing, where only the generation time of each fluid matters in the mixing process. In this case, the flow pattern is characterized by sequences of alternated slugs of two different gases flowing in a channel which are expected to be fully mixed at the exit of the mixer. Such flow pattern is obtained by generating alternate pulses of the two individual gas flows while keeping the overall flow rate constant. In this case, the mixing ratio depends on the ratio between the generation time of the first fluid and that of the second fluid.

Numerous studies in the literature have focused on the design, manufacture and validation of microfluidic chips for the homogenization of liquid solutions as reported in a recent review paper [23]. Conversely, far fewer studies have focused on obtaining homogeneous gas mixtures using microfluidic devices [24,25,26,27,28,29], with only one paper to date proposing a way of mixing axially heterogeneous gaseous flows [10] (see Table 1).

Here, Martin et al. reported a way of mixing such gaseous flows but in a non-flexible approach as the mixing device was designed for a given flow rate and a given mixing ratio. However, many processes require to either use several mixing ratios (chemical reactions) or varying flow rates (industrial processes, calibration of analytical devices) and often both of them. Therefore, another approach is needed for these applications. Table 1 summarizes the main technological solutions envisaged for obtaining homogeneous gas mixtures using microfluidic circuits at modular flow rates and mixing ratios. All these methods except for one have been used to mix continuous gas flows resulting in radial heterogeneity. Haas-Santo et al. [24] presented a very interesting way of mixing at flow rates up to 10,000 NmL·min^−1^, with a mixing time lower than 600 µs by using V-shaped microstructures that are splitting the flow into numerous sub-flows before recombining them. Another approach to create various concentrations of gases consists in injecting in two different inlet microchannels discrete concentrations of gases (pure N_2_ and pure O_2_) that are physically mixed in a three split and recombined levels of a tree-shaped micromixer [25]. In the nine outlet microchannels, the resulting concentrations of O_2_ varied from 0 to 100%. In another study, a gradient of O_2_ in N_2_ (0–100%) was obtained by using nine parallel inlet microchannels of three different O_2_ concentrations (0%, 50% or 100%). The gradient of concentration was achieved by diffusion through the PDMS walls in between the microchannels [26,27]. Here, the flow rates are smaller, ranging from 1 to 80 NmL·min^−1^, which corresponds to the investigated flow rates range. However, these methods cannot be used to generate a single chosen concentration. The gradient method makes it impossible to pick up one concentration inside the flow and the discrete concentrations method is limited by the number of concentrations being generated. This latter would also imply heavy wastes of gases, as one would pick only one channel for the chosen concentration and leave the others to exhaust. Finally, the basic T-shaped mixer studied by Huang et al. [28] shows homogeneity after only a few millimetres at 200 NmL·min^−1^ in a 550 µm × 125 µm (width × height) channel, showing the effectiveness of the diffusion process while mixing gases. However, this process is not as effective to balance the concentration of species in an axially heterogeneous flow, because the movement induced by the flow is opposed to diffusion between the two gases.

The objective of this work is then to develop a flexible mixing device made of microfluidic chips able to mix and homogenize pulsed gaseous flows at flow rates ranging from 1 to 100 NmL·min^−1^. To meet this challenge, the strategy is based on the creation of 4 parallel sub-flows entering buffer tanks patterned on a microchip in order to decrease the gas linear velocity and promote gas diffusion and mixing. The gases mixture being recombined passed the buffer tanks. The innovative part concerns the flexibility of the device with respect to the targeted gas flow rate. Indeed, to adapt to the needs of the user, a novel multi-stage modular system has been imagined and validated by numerical simulations in the range 1–100 NmL·min^−1^.

## 2. Materials and Methods 

### 2.1. Design of the Microfluidic Chip

The design of all the chips presented in this work have been made using the Autodesk Inventor software.

#### 2.1.1. Technical Constraints and Objectives

The objective of this study is to develop a design to mix pulses of a standard gas A delivered during *t_A_* = 1 s with pulses of pure air B delivering during *t_B_* = 9 s both at the inlet flow rate Q = 5 NmL·min^−1^, this case being considered as the extreme case in terms of pulses ratio (*t_A_*/(*t_A_*+*t_B_*)=1/10) and axial heterogeneity. Afterwards, several copies of this design may be used in series for mixing at higher flow rates up to 100 NmL·min^−1^ with the same values of *t_A_* and *t_B_.* The pressure at the device’s outlet and the temperature of the gas mixture are set to 1 atm and 23 °C respectively. Under these conditions at Q = 5 NmL·min^−1^, the volume of a pulse of standard gas A is V_A_ = 0.0832 NmL and the volume of a pulse of pure air B is V_B_ = 0.7488 NmL. Finally, the depth of the design is set to 1 mm, so that the mixer fits into a 5 cm × 10 cm rectangular chip.

#### 2.1.2. Strategy used to Design and Define the Mixing Microchip Pattern and Size

In the case of gases, diffusion coefficients are very high, in the order of 0.1 cm^2^·s^−1^ [30], compared to those of liquid, generally in the order of 1.10^−5^ cm^2^·s^−1^ [7,8]. This makes diffusion very effective at low flow rates, where the mass transfer by diffusion is significantly higher than that from advection. This is illustrated by the Peclet number *P_e_*, which is the ratio of the rate of advection to rate of diffusion and is given by the following equation [31]:

(1) $$P_{e}=\frac{L_{c}\times v}{D},$$

where *L_c_* is the characteristic length (in m), *v* is the average fluid’s velocity (in m·s^−1^) and *D* is the diffusion coefficient (in m^2^·s^−1^).

Increasing the flow rate increases the value of *P_e_* and then makes mass transfer by diffusion less efficient. Therefore, it is harder to achieve a fully homogeneous mixture at high flow rates by relying mainly on diffusion. The value of *P_e_* can be reduced by decreasing the fluid velocity. This can be done either by increasing the cross-section area through which the fluid passes or by splitting the flow.

However, increasing the cross-section is even more interesting because it also increases the contact area between two mixture layers [32], enhancing the effectiveness of the axial diffusion process. Splitting the flow into several channels of same cross-section decreases the velocity but it does not increase this contact area between layers. It is why it has been chosen to focus mainly on increasing the cross-section and creating an optimized design using buffer tanks.

The volume of this buffer tank must be at least equal to V_AB_ = V_A_ + V_B_ = 0.8333 NmL in order to properly mix every pulse of standard gas A with a pulse of pure air B at Q = 5 NmL·min^−1^. Nevertheless, this volume should be kept close to the volume V_AB_ to minimize the response time of the mixer. Therefore, the choice was arbitrarily focused on a buffer tank volume that was approximately 1.5 × V_AB_, that is, 1.2 mL.

**Figure 2:**
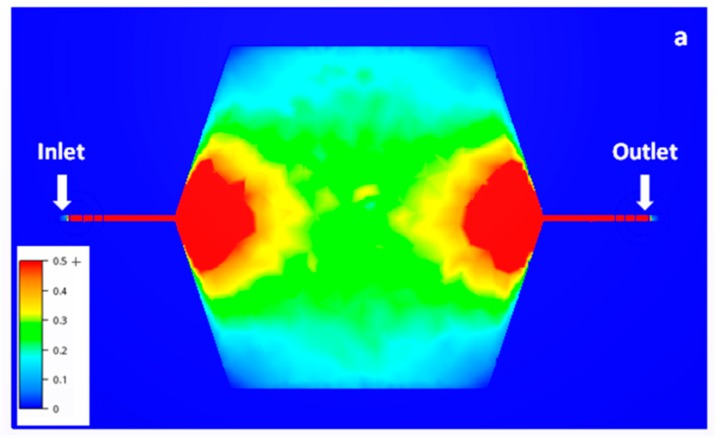

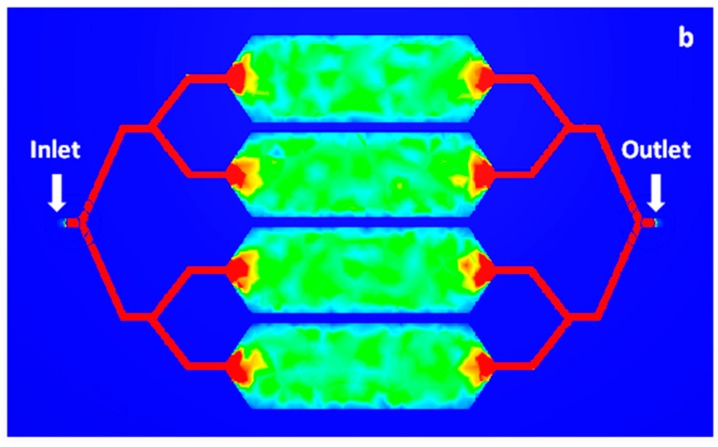
Velocity amplitude of a 3 cm long and 4 cm wide buffer tank (**a**) and of a set of 4 buffer tanks in parallel of 3 cm long and 1 cm wide each (**b**). The depth is fixed to 0.1 cm, the flow rate at the inlet is set to 5 NmL·min^−1^ and the pressure at the outlet is maintained at atmospheric pressure. The velocity scale is in cm·s^−1^ and is the same for insets. In red are represented areas with a velocity superior or equal to 0.5 cm·s^−1^. Both chips are 86 cm long and 49 cm wide. These simulations were performed using the Autodesk CFD (Computational Fluid Dynamics) software.

Given this latter volume and an imposed depth of 1 mm for the buffer tank, key variables become its width and its length. At a constant volume, increasing its length implies a decrease in its width and a reduction in the cross-section area, which would reduce the effectiveness of axial diffusion. On the other hand, increasing the width creates important variations in fluid velocity, leaving dead volumes at the edges of the buffer tank. Indeed, Figure 2a shows that there is a corridor with a stable mean velocity (in green) in the middle of the buffer tank. The velocity then decreases quickly down to almost 0 away from this corridor.

In order to increase the cross-section area without creating large dead volumes, it has been chosen to combine both aforementioned solutions and split the flow into numerous parallel buffer tanks before collecting all the fluid back into one single outlet. Figure 2b shows an example with 4 buffer tanks. The total cross-section area and the length of each of them are equal to those of the buffer tank presented in Figure 2a but the width of each buffer tank is 4 times smaller. In this way, the dead volumes have been greatly minimized. Of course, this final geometry may still be slightly improved in absence of size constraints in order to make the fluid velocity gradient even smoother.

**Figure 3:**
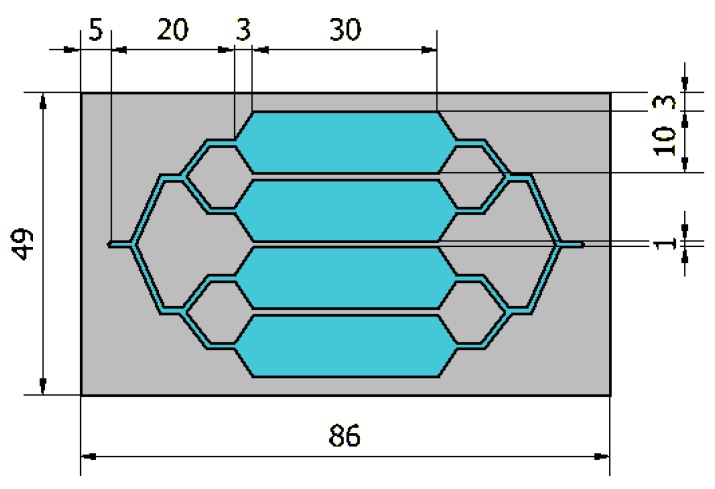
Dimensions of the optimized chip with 4 buffer tanks (given in mm).

Even though doubling several times the number of buffer tanks would have improved the mixer’s effectiveness, it has to be fitted into the 10 cm × 5 cm maximum size of the chip. In addition to these maximum dimensions, some convenience distances must be respected between the different elements for fabrication and assembly constraints, as illustrated in Figure 3:

- 5 mm between the inlet/outlet and the edges of the chip, to leave space for the fluidic connectors;
- 8 mm for every flow division: 4 mm for splitting and 4 mm for redirecting the flow into the right direction;
- 3 mm between channels and the walls of the chip;
- 1 mm between channels.

Because of these parameters, it would not be possible to increase the number of buffer tanks to 8, as it would exceed both target dimensions of 10 cm × 5 cm. Therefore, the 4 buffer tanks mixer (Figure 2b) is considered as the optimal system for the targeted application.

As illustrated in Figure 3, each buffer tank is 3 cm long and 1 cm wide which, considering the depth of 0.1 cm, makes the total volume of the 4 buffer tanks equal to 1.2 mL. Finally, the total volume of the single stage chip including channels and buffer tanks is 1.686 mL.

#### 2.1.3. Elaboration of the Multi-Stage Micromixer

**Figure 4:**
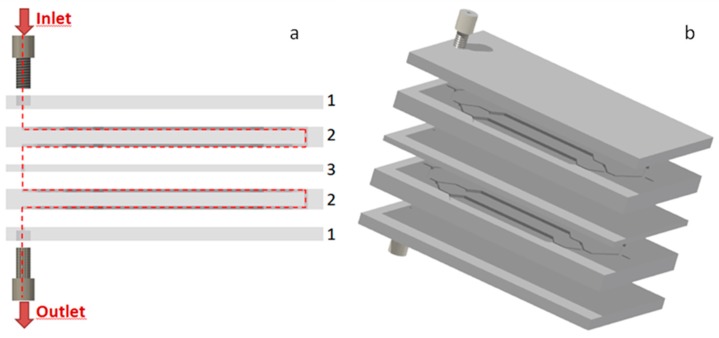
(**a**) Principle of the multi-stage design. The red line displays the path of the fluid flow. The design is made of 3 different chips: **1.** This chip is used to fix a 1/16” outer diameter connector serving as inlet or outlet ports. It is also the rooftop for microchannels; **2.** The chip presents 2 patterns containing 4 buffer zones each, one on each side of the chip. The outlet of one pattern is connected to the inlet of the other via a hole. **3.** An intermediate chip used as a rooftop for microchannels, separates the channels of the different chips number 2. (**b**) 3D schematics of the 4-stage mixer.

In order to homogenize gases mixtures with the same *t_A_* = 1 s and *t_B_* = 9 s at higher flow rates, several stages presenting the same pattern of 4 buffer tanks are connected in series. Thus, a flexible multi-stage micromixer composed of many identical chips can be easily obtained. As an example, Figure 4 represents a 4 stages micromixer. The first chip (1 on Figure 4), at the top and bottom of the assembly, is equipped with inlet and outlet fluidic connectors for 1/16” outer diameter tubings. The second chip (2 on Figure 4) presents a pattern of 4 buffer tanks on both sides. The third chip (3 on Figure 4) consists in a single hole to connect the outlet of one chip n°2 and the inlet of another chip n°2 and simultaneously to bond the lower part and the upper part of these two chips. Since only holes and two-dimensional patterns on each side of the stage are required, all these chips can be manufactured easily using a micro-milling machine. With this flexible design, it is possible to connect many mixing stages in series, with only one inlet and one outlet.

Considering that a single chip is made of 2 stages, the total volume of one double side chip (2 on Figure 4) including channels and buffer tanks is 3.372 mL, increasing the mixing capacity to mix pulses of gas A of *t_A_* = 1 s with pulses of gas B of *t_B_* = 9 s at a theoretical flow rate Q = 10 NmL·min^−1^.

### 2.2. Methodology for Simulation of the Gas Flow and Mixing

Several simulations have been made to determine the impact of the number of stages and the flow rate variations over the homogeneity and the time needed to achieve a steady state at the outlet. These simulations have been conducted for a compressible gas phase using the Autodesk CFD (Computational Fluid Dynamics) software and all the parameters used, and their values are listed in Table S1. The gases used for these simulations were air and formaldehyde. The temperature was considered constant all over the micromixer and equal to 23 °C. Reynolds number was calculated to be 110 at the inlet of the micromixer, which has the lowest section and highest flow rate (100 NmL·min^−1^) in the entire micromixer (1 mm^2^). This indicates that the flow is laminar in the whole system. In practice, A may represent a mixture of gas pollutant already very diluted in air (typically commercially available mixtures with concentrations in the range 0.1–100 ppm). However, the diffusion coefficient between formaldehyde and air (0.176 cm^2^·s^−1^) is very close to the self-diffusion coefficient of air (0.178 cm^2^·s^−1^), so the results would be identical.

The mixing of both gases at different points along the flow pathway was monitored using a scalar variable denoted S which is representative of gas A concentration in the mixture. For pure formaldehyde, the scalar was 1, while for pure air the scalar was 0. Therefore, a fully homogeneous mixture made of half formaldehyde and half air would have then got an average scalar of 0.5. In this study, mixing 1 s of formaldehyde at scalar 1 with 9 s of pure air at scalar 0 led to an average scalar of:

(2) $$S=\frac{t_{airA}}{t_{airA}+t_{airB}}=0.1.$$

The properties of the microchips’ material are those of Polyether Ether Ketone (PEEK), which is a non-reactive material used in many microfluidic applications, especially for tubings and fittings [33] and easily manufactured by micro-milling machines. An average reference mesh density of 757 nodes cm^−3^ was used for all the simulations. A comparison, for a flow rate of 25 NmL·min^−1^ and a pulses ratio of 1/10, with densities twice higher and lower has been made in order to confirm that the results achieved a satisfactory precision (Figure S1). Doubling the mesh density led to variations of the scalar value at the exit of the 4th mixing stage in the range of 2% compared to the reference mesh density. This was assumed to be a reliable test to validate the mesh density used for the simulations.

The diffusion coefficient between formaldehyde and air was calculated using Chapman-Enskog equation with a precision of about 8% according to Cussler [34]:

(3) $$D=\frac{1.86\times 10^{-3}\times T^{\frac{3}{2}}\times {\left(\frac{1}{M_{A}}+\frac{1}{M_{B}}\right)}^{\frac{1}{2}}}{p\times \sigma_{AB}^{2}\times \Omega },$$

where *D* is the diffusion coefficient (in cm^2^·s^−1^), *T* is the temperature (in K), *M_A_* and *M_B_* are the molecular weights of gas A and gas B respectively (in g·mol^−1^) and *p* is the pressure (in atm) *σ_AB_* is the average kinetic diameter between gases A and B, given by:

(4) $$\sigma_{AB}=\frac{\sigma_{A}+\sigma_{B}}{2},$$

where *σ_A_* and *σ_B_* are the kinetic diameters of gases A and B respectively. The collision integral Ω is a dimensionless quantity in the order of 1, which represents the interaction between both gases at a given temperature. Its value can be more precisely determined by calculating the Lennard-Jones potential between the two species [34]. Considering Ω = 1 and given the other parameters for gases A and B having the same properties than those of pure air, the diffusion coefficient used in these simulations was *D* = 0.178 cm^2^·s^−1^.

The values of both molecular weight *M* and kinetic diameter *σ* of air and several Volatile Organic Compounds (VOCs) are given in Table 2 [35,36,37], as well as their diffusion coefficient in air, resulting from these 2 parameters according to Equation (3). The calculated diffusion coefficients of VOCs in air vary in the range 0.071–0.176 cm^2^·s^−1^, the value of 0.071 cm^2^·s^−1^ being 2.5 times lower than air’s self-diffusion coefficient. Because this factor could have an impact on the effectiveness of the mixing device, a comparison was done between an air-formaldehyde mixture (*D* = 0.176 cm^2^·s^−1^) and an air-toluene mixture (*D* = 0.087 cm^2^·s^−1^). Even though other compounds such as o-Xylene and Naphthalene have lower diffusion coefficient in air, toluene was chosen for this comparison because it is one of the major indoor air pollutants along with formaldehyde.

Regarding the simulations, there were 2 boundary conditions at the inlet, which were the flow rate and the scalar of the gas entering the channels. The individual gases flow rates were varied depending on the scenario, while the scalar of the gas at the inlet is 1 for 1 s, then 0 for 9 s alternatively, simulating pulses of 1 s of formaldehyde alternating with pulses of 9 s of pure air. The boundary condition at the outlet was 1 atmosphere, since the set-up is supposed to operate at the atmospheric pressure.

The time step of the simulations was always 1 s, with 5 iterations per time step which allowed attaining a satisfactory convergence criterium (residual value of 1.0 × 10^−7^). This time step was verified to be low enough to guarantee reliable results by comparing the convergence criterium with that of a simulation performed with a time step of 0.1 s. The calculated scalar values were found to be the same within a difference lower than of 0.5%. However, for simulation time saving purposes, the time step of 1 s has been preferred for all simulations. For the same reasons, the results were saved only every 3 time’ steps (3 s).

## 3. Results

For every simulation, the first pulse of gas A entered the first stage at *t_0_* = 20 s. Because of the compressibility of the gas, pure gas B was generated during the first 20 s in order to reach a steady-state flow. Table 3 summarizes all the simulations that have been run, depending on the flow rate of the gaseous mixture and the number of mixing stages. Flow rates of 1, 5 and 10 NmL·min^−1^ have not been studied for 8 and 16 stages since (i) a satisfactory mixing has been reached with only 4 stages at these low flow rates, (ii) their corresponding response time at the outlet would have been become too long. Similarly, the 16 stages running at 25 NmL·min^−1^ was not studied either.

**Figure 5:**
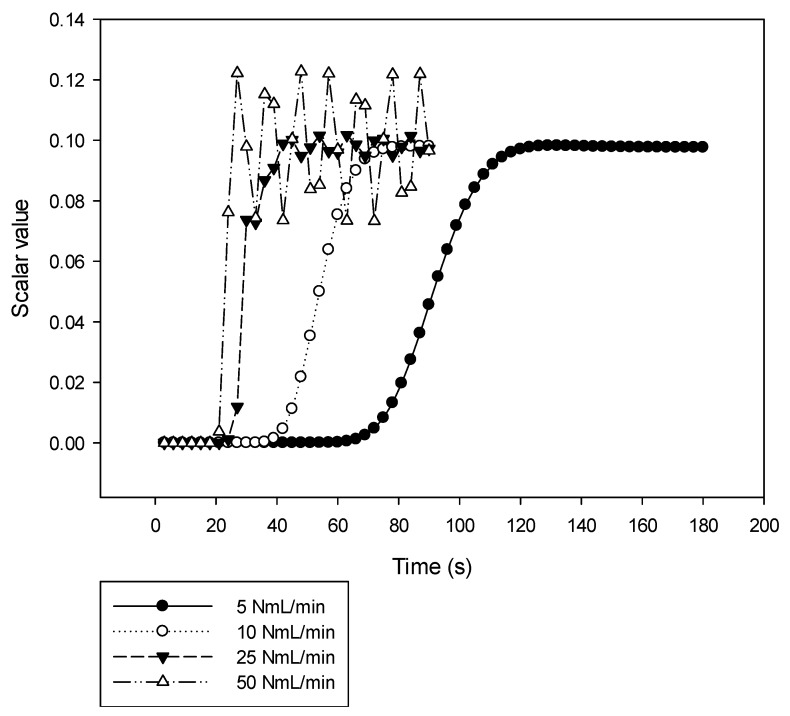
Effect of different flow rates on the variations of the scalar variable as a function of time at the exit of the last stage of a 4-stages micromixer.

**Figure 6:**
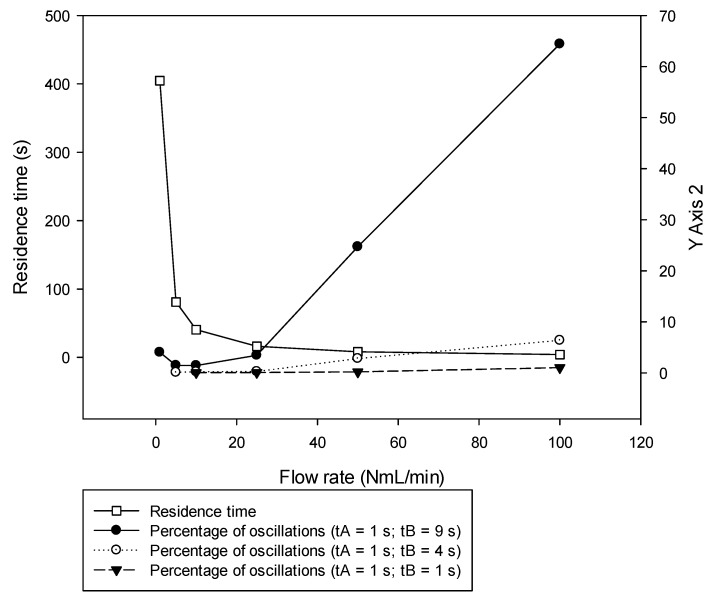
Residence time and percentage of oscillations around the targeted scalar 0.1 for flow rates ranging from 1 to 100 NmL·min^−1^ at the exit of the 4th mixing stage. Percentage of oscillations is given for *t_A_* = 1 s and *t_B_* = 9 s, 4 s and 1 s.

The scalar value for pure formaldehyde is monitored at the cross-section’s centre of the microchannel’s exit of the studied stages, as shown in Figure 5 for the 4th stage. At flow rates of 5 and 10 NmL·min^−1^, the scalar value slowly approaches the target value of 0.1 and stabilizes afterwards. At high flow rates, the scalar value oscillates around the targeted scalar value and the amplitude of these oscillations increases with the flow rate. For 50 NmL·min^−1^, the percentage of oscillation around the targeted scalar value is close to 20% while for flow rates from 25 NmL·min^−1^ and below, it is under 5%. This witnesses that the flow rate of 50 NmL·min^−1^ is too high to achieve a good mixing and indicates that more stages are required as illustrated in the following (see Figure 6).

To investigate whether the gas mixing operates in the buffer tanks of a stage or in the channels connecting the stages, the variations of the scalar value has been plotted versus time for a flow rate of 25 NmL·min^−1^ between (i) the inlet and outlet of one of the four parallel tank of the first stage (Figure S2a) and (ii) the exit of one tank of the first stage and the inlet of one tank of the second stage (Figure S2b). It is clearly seen that the mixing takes place exclusively in the tanks.

Figure 6 shows for the 4 stages device both variations of the residence time and the percentage of oscillation around the targeted value as a function of the flow rate for 3 different pulses ratios (1/10; 1/5; 1/2). As expected, it is observed that the residence time decreases with the flow rate while the percentage of oscillation at a pulses ratio of 1/10 sharply increases for flow rates higher than 10 NmL·min^−1^. Assuming that a satisfactory mixing is characterized by a percentage of oscillation lower than 5–6%, only flow rates up to 25 NmL·min^−1^ can be considered for the highest pulses ratio investigated, that is 1/10. For lower pulses ratios, since the axial diffusion pathway for gas mixture homogenization is reduced, the flow rate to reach the targeted percentage of oscillations is increased. In other terms, for a given flow rate, the lower the pulse flow rate, the lower is the percentage of oscillations and better is the mixing. For example, at 100 NmL·min^−1^, the percentage of oscillations reaches 64.4%, 6.4% and 1.0% for a pulses’ ratio of 1/10, 1/5 and 1/2 respectively and 24.7%, 2.8% and 0.2% at 50 NmL·min^−1^.

The percentage of oscillations for different number of stages is plotted versus the flow rate in the range 1–100 NmL·min^−1^ (see Figure S3). For a given flow rate and a pulses ratio of 1/10, the percentage of oscillations decreases with the number of stages since a longer residence time promotes a better gas mixing.

**Figure 7:**
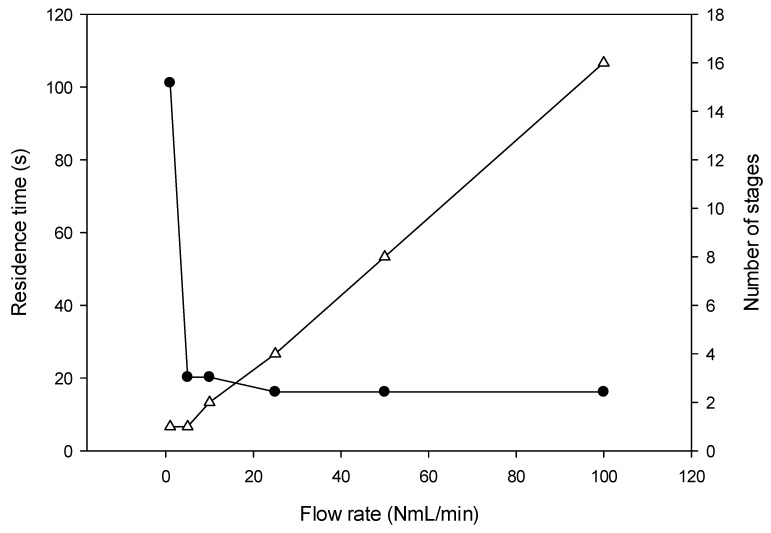
Number of stages (Δ) required for the oscillations of the scalar value at the micromixer’s exit to be lower than 5% of the targeted value 0.1 at flow rates ranging from 1 to 100 NmL·min^−1^. Variations of the residence time (•) with the flow rate for the corresponding number of stages required. At 100 NmL·min^−1^, the 5% threshold is not reached: the percentage of oscillations is 6.1% after the 16th stage of mixing.

The number of stages requested to achieve a percentage of oscillation lower than 5–6% as a function of the flow rate and the subsequent residence time is represented in Figure 7 for a pulses ratio of 1/10. Above 1 NmL·min^−1^, the number of required stages increases linearly (R^2^ = 0.99) from 1 to 16 for flow rate ranging between 5 and 100 NmL·min^−1^, respectively whereas the residence time stays almost constant in the range 16.2–20.2 s. It means that whatever the flow rate desired, it is possible to adjust the number of stages to achieve a satisfactory mixing with a constant residence time of around 16–20 s. Furthermore, this number of stages will decrease in case of a higher pulses’ ratio, for example, 1/5 or 1/2.

Finally, a comparison between an air-formaldehyde mixture, an air-toluene mixture and air-air has been made in order to investigate the effect of the diffusion coefficient on the mixing’s efficiency.

**Figure 8:**
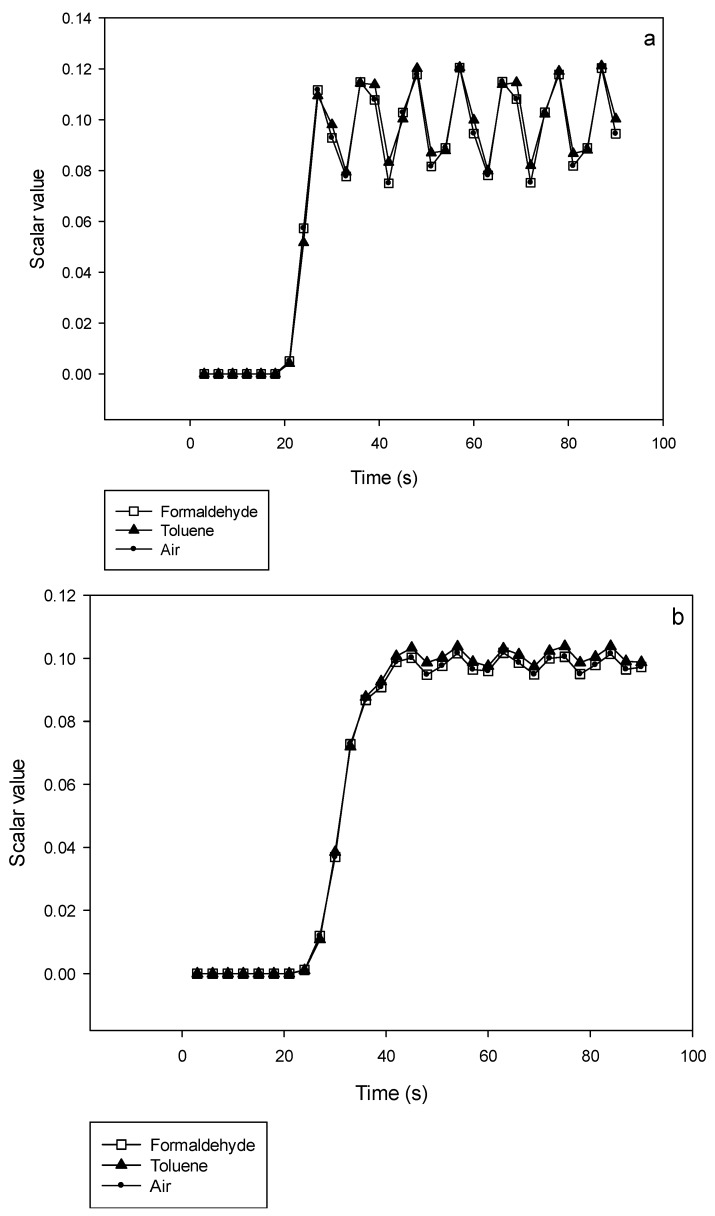
Comparison of the mixing’s efficiency between formaldehyde, toluene and air being mixed with pure air at 25 NmL·min^−1^. Variations of the scalar value with time at the exit of a 2-stages (**a**) or 4-stages (**b**) device, illustrating that there is almost no time response difference between formaldehyde, toluene and air.

In Figure 8a,b, the scalar value is monitored at the exit of a 2-stages and a 4-stages device respectively for different gas mixtures where B is pure air (*t_B_* = 9 s) and A is either pure formaldehyde, pure toluene or pure air (*t_A_* = 1 s) at a given flow rate of 25 NmL·min^−1^. At the 2 stages device’s exit, the amplitude of the oscillations is close to 20% of the targeted scalar value whatever the nature of gas B, while it reaches 5 % for the 4-stages device.

One may have expected that the gas mixing efficiency would have been dependent on the diffusion coefficient in air for the different studied gases, that is *D*_HCHO_ = 0.176 cm^2^·s^−1^; *D_toluene_* = 0.087 cm^2^·s^−1^ and *D*_air_ = 0.178 cm^2^·s^−1^. However, the simulations shown in Figure 8 demonstrate that differences between those 3 gases are already very small after 2 stages and negligible for the 4 stages device. Such results indicate the strong efficiency of the mixing device developed in this work.

Because there is no difference, neither in stability nor in response time, between air, formaldehyde and toluene, it can be assumed that the simulations of this work can be used for all the VOCs listed in Table 2, with diffusion coefficients ranging from 0.071 to 0.176 cm^2^·s^−1^.

In case of gas A is already composed of a diluted gaseous component in air, the same behaviour is expected as that observed for a pure gas A, which permits to potentially generate extremely low gas concentrations of A. 

Other alternative solution to dilute more the gas A consists in increasing the ratio between times *t_A_* and *t_A_* + *t_B_*, for example, *t_A_*= 0.1 s and *t_B_* = 9.9 s. For a given flow rate of 5 NmL·min^−1^, the percentage of oscillation decreases with number of stages: 44.7% (1 stage); 10.1% (2 stages); 3.4% (3 stages) and 0.5% (4 stages). As illustrated in Figure S4, the corresponding residence time is increased from 16s to around 48 s and 64 s for 3 and 4 stages, respectively.

## 4. Discussion

As aforementioned, few experimental studies have investigated the mixing of 2 gases in microfluidic devices (see Table 1) by the radial approach. Only one study [10] refers to the axial approach and was a numerical investigation like in the present work.

As presented in Table 1, most of the microchips used for mixing gases have a fixed design [10,25,26,29], meaning that either the flow rate or the mixing ratios or both, cannot be changed using a single microchip. This is an issue for these devices where many applications require on line and rapid parameters changes. For instance, this is the case for analytical calibration instruments or during the generation of a protective gaseous mixture during welding. Conversely, the technical flexible solution presented in this work offers to the user the possibility to have a modular system where mixing ratios and total gas flow rates can easily vary in the ranges 1/10–9/10 and 1–100 NmL·min^−1^, respectively.

Most of gas mixing studies have been based on a radial approach where they required mixing times ranging between 4 and 24 s (see Table 1) [25,26,27]. The technical solution proposed by Polinkovski et al. [25] achieved homogeneity after 4 s using a microchannels network to generate discrete concentrations of O_2_ in N_2_. Two other microchips, used to generate O_2_ concentrations for cell culture, exhibited mixing time of 20–24 s [26,27]. In addition, most of them [26,27] proposed a fixed design limiting any parameters changes. The proposed modular device allows the selection of the right number of stages needed for a given flow rate in order to obtain a response time of 20 s whatever the flow rate with oscillations of the targeted concentration lower than 5% (Figure 7).

Haas-Santo et al. [24] and Huang et al. [28] have developed very fast mixers for radial heterogeneity, with mixing times down to 0.6 and 3.7 ms, respectively. The former allows efficient gas mixing at very high flow rates up to 10,000 NmL·min^−1^, while the latter operates at a range of flow rates (5–250 NmL·min^−1^) close to the range of interest (1–100 NmL·min^−1^). However, these devices require very accurate and expensive techniques in clean rooms to manufacture microchips integrating small microchannels in the order of 100 µm × 100 µm and 550 µm × 125 µm, respectively [24,28]. On the contrary, the chip has larger channels of 1 mm × 1 mm and tanks of 10 mm × 1 mm, so that it can be thus easily manufactured using a micromilling machine. Furthermore, these two devices [24,28] could never have been used for mixing pulsed gas flows since the small microchannels cross section would have induced the generation of long plugs of gases to be mixed; which in turn would have required a long mixing time. This fully justifies the development of a specific micromixer for the axial mixing of gases.

Moreover, the device allows response times of a few tens of seconds, by accommodating the number of stages according to the total flow rate, conversely to the approximately 2 min reported by the other axial numerical diffusion study [10], not to mention its lack of flexibility.

## 5. Conclusions

A new axial gas pulses multi-stage micromixer has been designed to allow an efficient mixing between two pulses of different gases (A into B), easily allowing the generation of different gas concentrations for many applications such as cell culture or analytical calibration.

Once the dimensions and the geometry of a single stage determined and optimized, modelling was performed by CFD and has demonstrated that the device allows response times of few tens of seconds. This response time was obtained by adjusting the number of stages (1 to 16) according to the total gas flow rate (1 to 100 NmL·min^−1^) to reach 94–95% of the theoretical mixing ratio calculated by the pulses ratio; and corresponds to the residence time of the micromixer. As an example, a single stage of 4 buffer tanks achieves homogeneity at a flow rate of 5 NmL·min^−1^ within 20 s for a pulses ratio of 10% (*t_A_* = 1 s and *t_B_* = 9 s), while 16s and 16 stages are required for a flow rate of 100 NmL·min^−1^.

If the gas A is already diluted in gas B at a concentration of 100 ppm (commercial product), then the targeted concentrations of A at the device exit can vary in the range 10–90 ppm for a given flow rate by adjusting the A pulses ratio, 1/10 to 9/10 respectively. 1/100 dilution could be also achieved by combining pulses of *t_A_* = 0.1 s and *t_B_* = 9.9 s if it is acceptable to have a longer mixing time. In practice, pulses times can be easily controlled by software using electronic valves to become automatic and user friendly. Thus, the proposed device could serve for calibration purposes for instance.

## Figures and Tables

**Table 1:** Technological solutions used to obtain homogeneous gas mixtures in microfluidic devices

Short Description/Technology Used	Approach	Applications	Type of Heterogeneity	Total Flow Rate (NmL·min^−1^)	Mixing Time (s)	Microchip Design	Reference
Fluids collision inducing oscillations for mixing liquids or gases	Experimental (liquids)	Fuel technology	Radial	-	-	Fixed	Tesař et al., 2000 [24]
Multilamination by using V-shaped microstructures	Experimental	Chemical reaction engineering	Radial	1000–10,000	6 × 10^−4^	Modular	Haas-Santo et al., 2005 [25]
Microchannels network generating discrete concentrations of O_2_ in N_2_	Experimental	Biotechnology, cell culture	Radial	16.2	4	Fixed	Polinkovsky et al., 2009 [26]
Mixing of 9 gas flows at different O_2_ concentrations to create O_2_ concentration gradients	Experimental	Cell culture	Radial	108	24	Fixed	Adler et al., 2009 [27]
Diffusion between parallel flow channels through PDMS layer to create an O_2_ concentration gradient	Experimental	Cell culture	Radial	80	20	Modular	Lo et al., 2010 [28]
Splitting of the flow between inlet and outlet chambers, followed by a buffer tank	Modelling	Calibration gases generation	Axial	-	120	Fixed	Martin et al., 2012 [10]
Basic T-shaped mixer	Experimental	Microcombuster, fuel technology	Radial	5–250	3.7 × 10^−3^	Modular	Huang et al., 2017 [29]
Multistage mixing microchips for pulsed gas flow	Modelling	Gas mixture generation	Axial	1–100	20	Modular	This work

**Table 2:** Diffusion coefficient of several gases in air at 1 atm and 23 °C.

Gas	Molecular Weight (g·mol^−1^)	Kinetic Diameter *σ* (Å)	Diffusion Coefficient in Air (cm²·s^−1^)
Air	28.97	3.71 [8]	0.178
Formaldehyde (HCHO)	30.03	3.73 [35]	0.176
Acetaldehyde (CH_3_CHO)	44.05	7.27 [36]	0.074
Benzene (C_6_H_6_)	78.11	5.85 [37]	0.089
Toluene (C_7_H_8_)	92.14	5.85 [37]	0.087
Ethylbenzene	106.17	6.00 [37]	0.083
p-Xylene	106.16	5.85 [37]	0.086
m-Xylene	106.16	6.80 [37]	0.071
o-Xylene	106.16	6.80 [37]	0.071
Naphthalene	128.17	6.20 [37]	0.078

**Table 3:** Presentation of the simulations realized at different flow rates varying between 1 and 100 NmL·min^−1^ and numbers of stages in the range 1–16. Configurations marked with a ✓ have been simulated, while configurations marked with a ✕ have not been studied. The total microfluidic circuit volume of the stages set is also calculated and mentioned on the right.

Flow Rate (NmL·min^−1^)	1	5	10	25	50	100	Total Volume (mL)
Number of Stages							
1	✓	✓	✓	✓	✓	✓	1.686
2	✓	✓	✓	✓	✓	✓	3.372
3	✓	✓	✓	✓	✓	✓	5.058
4	✓	✓	✓	✓	✓	✓	6.744
8	✕	✕	✕	✓	✓	✓	13.488
16	✕	✕	✕	✕	✓	✓	26.976

## Supplementary Materials

The following are available online at https://www.mdpi.com/2072-666X/10/3/205/s1, Table S1: Parameters of the simulations, Figure S1: Comparison of the results for mesh densities equal to 0.5, 1 and 2 times the reference mesh density with the same mixing parameters (Q = 25 NmL·min^−1^ and *t_A_*/(*t_A_* + *t_B_*) = 1/10) at the exit of the 4th mixing stage, Figure S2: Scalar values variations with respect to time between the inlet and the outlet of a tank from the first stage (a) and between the outlet of this tank and the inlet of a tank from the second stage (b), Figure S3: Scalar variations at the exit of different numbers of mixing stages with respect to the flow rate. The variations are presented as a +/- percentage of the targeted scalar 0.1, Figure S4: Scalar value after 1 to 4 stages at a flow rate of 5 NmL·min^−1^ for a pulses ratio of 1/100.

## References

[1] Robbins P.A., Swanson G.D., Micco A.J., Schubert W.P. (1982). A fast gas-mixing system for breath-to-breath respiratory control studies. J. Appl. Physiol. Respir. Environ. Exerc Physiol..

[2] Dantas H.V., Barbosa M.F., Moreira P.N.T., Galvão R.K.H., Araújo M.C.U. (2015). An automatic system for accurate preparation of gas mixtures. Microchem. J..

[3] Continuous Flow Type Gas Blending Facility Used for Autonomous and System Diving—ScienceDirect. https://www.sciencedirect.com/science/article/pii/S1876610217311748.

[4] Fletcher G.C., Summers G., Corrigan V.K., Johanson M.R., Hedderley D. (2005). Optimizing Gas Mixtures for Modified Atmosphere Packaging of Fresh King Salmon (Oncorhynchus tshawytscha). J. Aquat. Food Prod. Technol..

[5] Hood M.E. (1951). Gas Mixing Device for Draught Beer Dispensing. U.S. Patent.

[6] Mvola B., Kah P. (2017). Effects of shielding gas control: Welded joint properties in GMAW process optimization. Int. J. Adv. Manuf. Technol..

[7] Shmelev V.M., Nikolaev V. (2011). Propane conversion in a chemical compression reactor. Russ. J. Phys. Chem. B.

[8] Zethræus B., Adams C., Berge N. (1992). A simple model for turbulent gas mixing in CFB reactors. Powder Technol..

[9] Christensen P.L., Nielsen J., Kann T. (1992). Methods to produce calibration mixtures for anesthetic gas monitors and how to perform volumetric calculations on anesthetic gases. J. Clin. Monit. Comput..

[10] Martin N.A., Goody B.A., Wang J., Milton M.J.T. (2012). Accurate and adjustable calibration gas flow by switching permeation and diffusion devices. Meas. Sci. Technol..

[11] Rosenberg E., Hallama R.A., Grasserbauer M. (2001). Development and evaluation of a calibration gas generator for the analysis of volatile organic compounds in air based on the injection method. Fresenius J. Anal. Chem.

[12] Monsé C., Broding H., Hoffmeyer F., Jettkant B., Berresheim H., Brüning T., Bünger J., Sucker K. (2010). Use of a Calibration Gas Generator for Irritation Threshold Assessment and As Supplement of Dynamic Dilution Olfactometry. Chem. Sens..

[13] Pérez Ballesta P., Baldan A., Cancelinha J. (1999). Atmosphere Generation System for the Preparation of Ambient Air Volatile Organic Compound Standard Mixtures. Anal. Chem..

[14] Ricker N.L., Muller C.J., Craig I.K. (2012). Fuel gas blending benchmark for economic performance evaluation of advanced control and state estimation. J. Process. Control..

[15] Safdar M., Jänis J., Sánchez S. (2016). Microfluidic fuel cells for energy generation. Lab Chip.

[16] Seong G.H., Crooks R.M. (2002). Efficient Mixing and Reactions within Microfluidic Channels Using Microbead-Supported Catalysts. J. Am. Chem. Soc..

[17] Shang L., Cheng Y., Zhao Y. (2017). Emerging Droplet Microfluidics. Chem. Rev..

[18] Zhu P., Wang L. (2016). Passive and active droplet generation with microfluidics: A review. Lab. Chip.

[19] Christopher G.F., Anna S.L. (2007). Microfluidic methods for generating continuous droplet streams. J. Phys. D Appl. Phys..

[20] Baroud C.N., Willaime H. (2004). Multiphase flows in microfluidics. C. R. Phys..

[21] Zhao C.-X., Middelberg A.P.J. (2011). Two-phase microfluidic flows. Chem. Eng. Sci..

[22] Lee C.-Y., Chang C.-L., Wang Y.-N., Fu L.-M. (2011). Microfluidic Mixing: A Review. Int. J. Mol. Sci..

[23] Suh Y.K., Kang S. (2010). A Review on Mixing in Microfluidics. Micromachines.

[24] Haas-Santo K., Pfeifer P., Schubert K., Zech T., Hönicke D. (2005). Experimental evaluation of gas mixing with a static microstructure mixer. Chem. Eng. Sci..

[25] Polinkovsky M., Gutierrez E., Levchenko A., Groisman A. (2009). Fine temporal control of the medium gas content and acidity and on-chip generation of series of oxygen concentrations for cell cultures. Lab. Chip.

[26] Adler M., Polinkovsky M., Gutierrez E., Groisman A. (2010). Generation of oxygen gradients with arbitrary shapes in a microfluidic device. Lab Chip.

[27] Lo J.F., Sinkala E., Eddington D.T. (2010). Oxygen gradients for open well cellular cultures via microfluidic substrates. Lab Chip.

[28] Huang C.-Y., Wan S.-A., Hu Y.-H. (2017). Oxygen and nitrogen gases mixing in T-type micromixers visualized and quantitatively characterized using pressure-sensitive paint. Int. J. Heat Mass Transf..

[29] Tesař V.R., Tippetts J., Low Y.-Y. Oscillator Mixer for Chemical Microreactors. Proceedings of the 9th International Symposium on Flow Visualization.

[30] Wilke C.R., Lee C.Y. (1955). Estimation of Diffusion Coefficients for Gases and Vapors. Ind. Eng. Chem..

[31] Squires T.M., Quake S.R. (2005). Microfluidics: Fluid physics at the nanoliter scale. Rev. Mod. Phys..

[32] Faanes A., Skogestad S. (2000). A systematic approach to the design of buffer tanks. Comput. Chem. Eng..

[33] Tsao C.-W. (2016). Polymer Microfluidics: Simple, Low-Cost Fabrication Process Bridging Academic Lab Research to Commercialized Production. Micromachines.

[34] Cussler E.L. (1997). Diffusion: Mass Transfer in Fluid Systems.

[35] (PDF) Adsorption of Low-Concentration Formaldehyde from Air by Silver and Copper Nano-Particles Attached on Bamboo-Based Activated Carbon. https://www.researchgate.net/publication/271305127_Adsorption_of_Low-Concentration_Formaldehyde_from_Air_by_Silver_and_Copper_Nano-Particles_Attached_on_Bamboo-Based_Activated_Carbon.

[36] Gauf A., Navarro C., Balch G., Hargreaves L.R., Khakoo M.A., Winstead C., McKoy V. (2014). Low-energy elastic electron scattering by acetaldehyde. Phys. Rev. A.

[37] Weng Y., Qiu S., Ma L., Liu Q., Ding M., Zhang Q., Zhang Q., Wang T. (2015). Jet-Fuel Range Hydrocarbons from Biomass-Derived Sorbitol over Ni-HZSM-5/SBA-15 Catalyst. Catalysts.

