# Supplementary material for: Design of a Novel Axial Gas Pulses Micromixer and Simulations of its Mixing Abilities via Computational Fluid Dynamics
Source: Micromachines (Basel). 2019 Mar 23;10(3):205. doi: 10.3390/mi10030205 (PMC6471047; doi:10.3390/mi10030205)
Supplement: Supplementary file 1 [file micromachines-10-00205-s001.pdf]

micromachines

Supplementary Materials: Design of A Novel Axial Gas Pulses Micromixer and Simulations of Its Mixing Abilities via Computational Fluid Dynamics

Florian Noël [1,2,3], Christophe A. Serra [3] and Stéphane Le Calvé [1,2,*]

Table S1. Parameters of the simulations.

	Parameter	Value	Unit
Reference Mesh Density		757[a]	Nodes cm^{-3}
Initial Conditions	Initial scalar value throughout the micromixer	0	-
Boundary Conditions	Flow rate at the inlet	From 1 to 100	NmL min^{-1}
	Scalar value at the inlet	1 for gas A<br>0 for gas B	-
	Pressure at the outlet	1	atm
	Operating temperature	23	°C
Time step		1	s
Iterations per time step		5	-

[a] The reference mesh density is reported as an average value since higher mesh densities were employed in the microchannels.

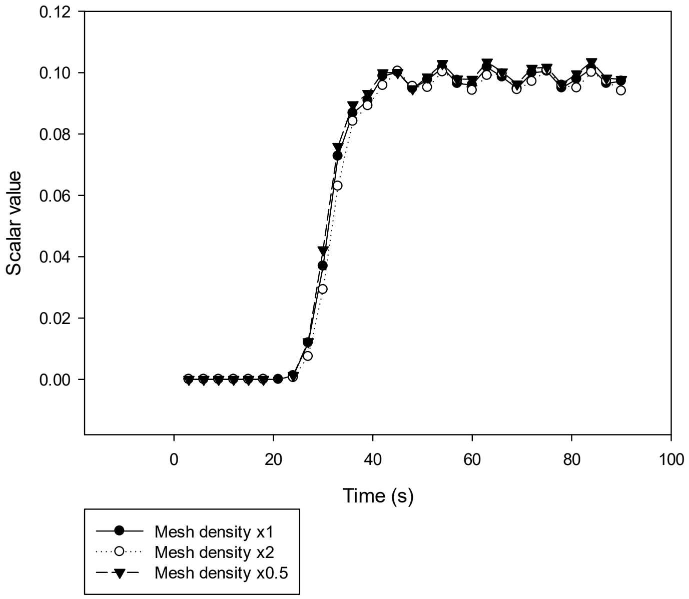

Figure S1. Comparison of the results for mesh densities equal to 0.5, 1 and 2 times the reference mesh density with the same mixing parameters (Q = 25 NmL·min^{-1} and $t_A/(t_A + t_B)$ = 1/10) at the exit of the 4th mixing stage.

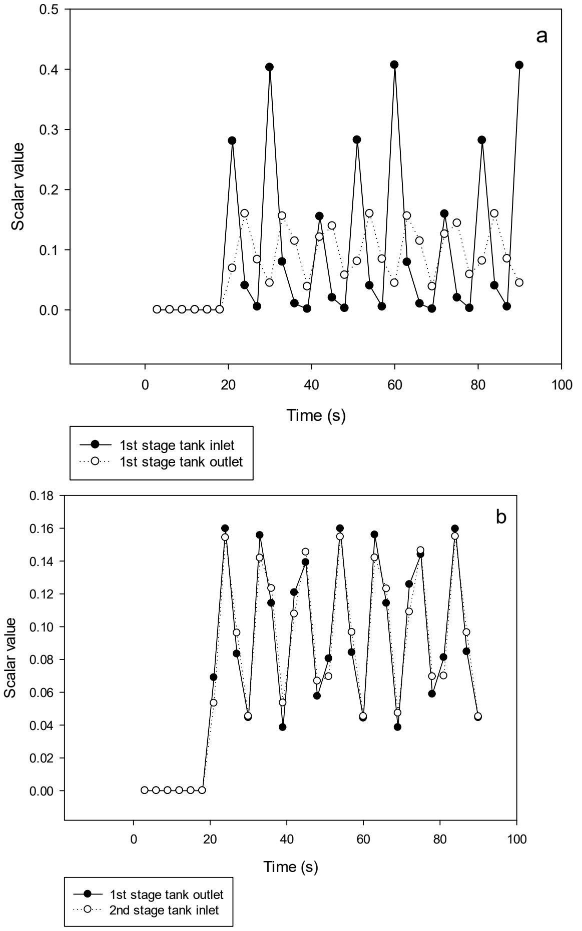

Figure S2. Scalar values variations with respect to time between the inlet and the outlet of a tank from the first stage (**a**) and between the outlet of this tank and the inlet of a tank from the second stage (**b**).

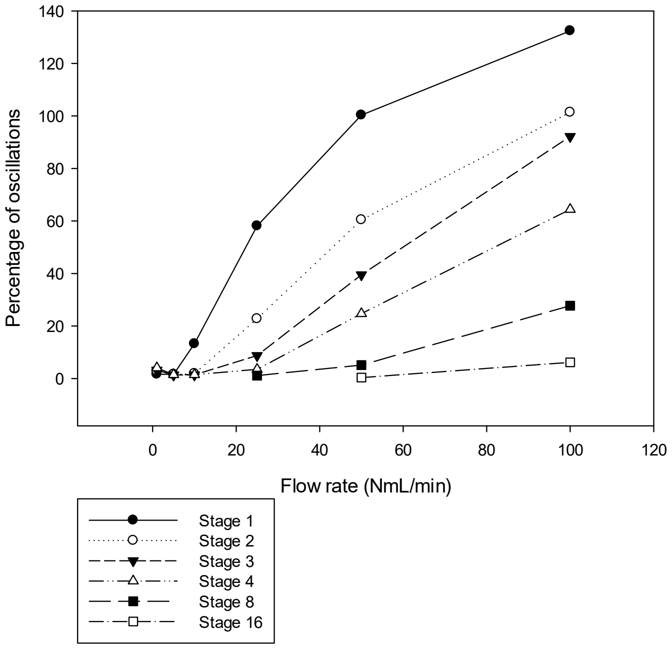

Figure S3. Scalar variations at the exit of different numbers of mixing stages with respect to the flow rate. The variations are presented as a +/- percentage of the targeted scalar 0.1.

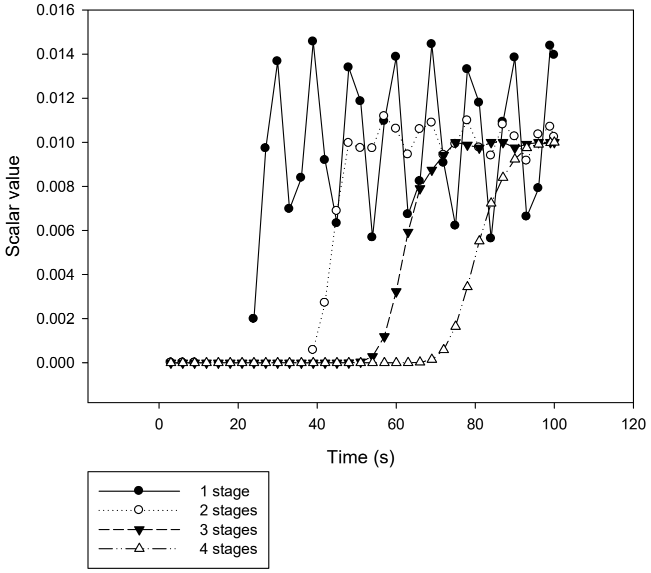

Figure S4. Scalar value after 1 to 4 stages at a flow rate of 5 NmL·min^{-1} for a pulses ratio of 1/100.
